# The effects of volatile anesthetics and propofol in patients undergoing off-pump coronary artery bypass grafting: a systematic review and meta-analysis

**DOI:** 10.3389/fcvm.2023.1271557

**Published:** 2023-11-15

**Authors:** Chenghong Zhang, Changlin He, Zhengwei Chen, Xin Chen, Junjun Qin, Yuhui Xu, Jiasen Ma

**Affiliations:** ^1^ School of Medicine, Hangzhou Normal University, Hangzhou, China; ^2^ Department of Anesthesiology, Affiliated Hospital of Hangzhou Normal University, Hangzhou, China

**Keywords:** volatile anesthetic, sevoflurane, desflurane, isoflurane, propofol, coronary artery bypass grafting, cardiac troponins

## Abstract

**Background:**

Studies investigating the cardioprotective effect of volatile anesthetics on cardiac troponins in off-pump coronary artery bypass grafting (OPCAB) surgery remain controversial. This current study was conducted to systematically evaluate the impact of volatile anesthetics and propofol on patients undergoing OPCAB surgery.

**Methods:**

A computerized search of electronic databases was conducted up to July 21, 2023, to identify relevant studies using appropriate search terms. The primary outcomes of interest were the levels of myocardial injury biomarkers (e.g., cTnI, cTnT), while secondary outcomes included extubation time, length of ICU stay, 30-day mortality, transfusion and thrombosis, and postoperative recovery, which were compared between two anesthesia techniques.

**Results:**

A search of databases produced 14 relevant studies with a combined total of 703 patients. Among them, 355 were allocated to the volatile anesthetics group and 348 to the propofol group. Our study reveals a statistically significant reduction in myocardial injury biomarkers among patients who received volatile anesthetics compared to those who received propofol (*P* < .001). Subgroup analysis showed that patients using sevoflurane had lower postoperative cardiac troponins levels compared to propofol (*P* = .01). However, desflurane and isoflurane currently have no significant advantage over propofol (all *P* > 0.05). There was no significant difference in postoperative mechanical ventilation time, length of ICU stay, and mortality between the two groups (all *P* > 0.05).

**Conclusions:**

This study suggested that volatile anesthetics, specifically sevoflurane, in adult OPCAB surgery provide a better cardioprotective effect than propofol.

**Systematic Review Registration:**

PROSPERO (CRD42023444277).

## 1. Introduction

Coronary artery bypass grafting (CABG) surgery is a crucial approach to the management of coronary heart disease. Patients who require this procedure often present with poor cardiac function, advanced age, and comorbidities, which increase the risk of perioperative myocardial injury (PMI) and compromise clinical outcomes. Therefore, protecting the vulnerable cardiac during CABG surgery remains a critical area of investigation. Volatile anesthetics, including isoflurane, sevoflurane, and desflurane, have demonstrated myocardial protection against acute ischemia-reperfusion injury (IRI) by reducing infarct size in animal models (1, 2). A meta-analysis has shown that inhalational anesthesia may decrease postoperative myocardial calcium protein levels in on-pump CABG surgery patients (3). However, the impact of volatile anesthetics on cardioprotection in patients undergoing OPCAB surgery remains to be explored.

Cardiac biomarker profiling can help evaluate the degree of myocardial damage during cardiac surgery in the perioperative period. A considerable number of clinical trials have used serum cardiac enzymes (creatine kinase type MB (CK-MB), troponin T (cTnT), and troponin I (cTnI)) to quantify the extent of PMI sustained during surgery. Additionally, the severity of the biomarker elevation can assist in predicting both short-term perioperative mortality rates and long-term patient mortality risk (4, 5). We have undertaken a systematic review of peak serum cardiac troponin levels in those clinical studies that have investigated the cardioprotective effect of volatile anesthetics in OPCAB surgery using PMI as the endpoint.

## 2. Materials and methods

### 2.1. Ethical approval

Ethical approval was not necessary because this study was a systemic review of previously published literature.

### 2.2. Search strategy

The protocol of the current meta-analysis was published in PROSPERO with the registration number CRD42023444277. We conducted a systematic literature search for all relevant prospective randomized clinical trials and retrospective studies in Chinese and English languages. Relevant trials between 2003 and 2017 were obtained from the following sources: electronic databases [Medline and Excerpta Medica (EMBASE) EMBASE], the Cochrane Controlled Trial Register, and the Chinese BioMedical Literature & Retrieval System. All database search was updated on July 21st, 2023.

### 2.3. Inclusion and exclusion criteria

Eligible prospective randomized controlled trials (RCTs) and retrospective studies should include OPCAB surgery patients randomized into volatile anesthetic (including sevoflurane, isoflurane, and desflurane) as compared to propofol. Halothane and enflurane studies were excluded because they were considered not to reflect current clinical practice. We excluded studies published as meta-analysis, guidelines, expert opinions, case reports, protocol or abstracts, animal studies, duplicate publications, and studies lacking outcome data. Two authors (ZCH and MJS) independently assessed all identified studies for eligibility. Disagreement was resolved by consensus or the opinion of a third reviewer.

### 2.4. Study quality assessment

Two authors independently assessed the risk of bias in each included study, following strict accordance with the Cochrane Handbook for systematic reviews of interventions. We utilized the risk of bias assessment table provided by the same publication. The Cochrane risk of bias assessment tool encompasses the domains of sequence generation (selection bias), allocation sequence concealment (selection bias), blinding of participants and personnel (performance bias), blinding of outcome assessment (detection bias), incomplete outcome data (attrition bias), selection outcome reporting (reporting bias), and other potential sources of bias. The authors' judgments were classified as containing "low risk", "high risk", or "unclear risk" of bias.

### 2.5. Outcomes and data abstraction

To ensure consistency with previous studies in reflecting the extent of PMI, we standardized cTnT to cTnI. For studies that only reported cTnT levels, the levels of cTnI were calculated using a conversion factor of 3.076 (2/0.65) based on the ratio of their respective upper limits for the reference ranges (3). We included the peak postoperative cTnI levels reported by each group in the final meta-analysis. In three trials where continuous outcomes were reported as median and range, mean and standard deviation were estimated using the O'Rourke method (6).

We also conducted a meta-analysis of postoperative extubation time and length of ICU stay time for two groups. Due to insufficient data, no analysis was performed on the following outcomes in both patient groups: myocardial infarction, heart failure, 30-day mortality, perioperative, and reoperation.

### 2.6. Statistical analysis

Meta-analysis was performed to evaluate the outcomes among the finally included studies. The continuous data were presented as the weighted mean difference and a 95% confidence interval. The statistical heterogeneity of each outcome was evaluated, and a random-effects or fixed-effects model was selected based on the presence or absence of significant heterogeneity (*I*^2^ > 50%). Sensitivity analyses were performed to examine the effects of the statistical model on the estimated treatment effects. All statistical analyses were performed using the Rev Man 5.4 (Cochrane Collaboration, Oxford, UK). Statistical significance was defined as *P* < .05.

## 3. Results

### 3.1. Search results

In this study, a total of 636 related articles were evaluated and then narrowed down for meta-analysis.

**Figure 1:**
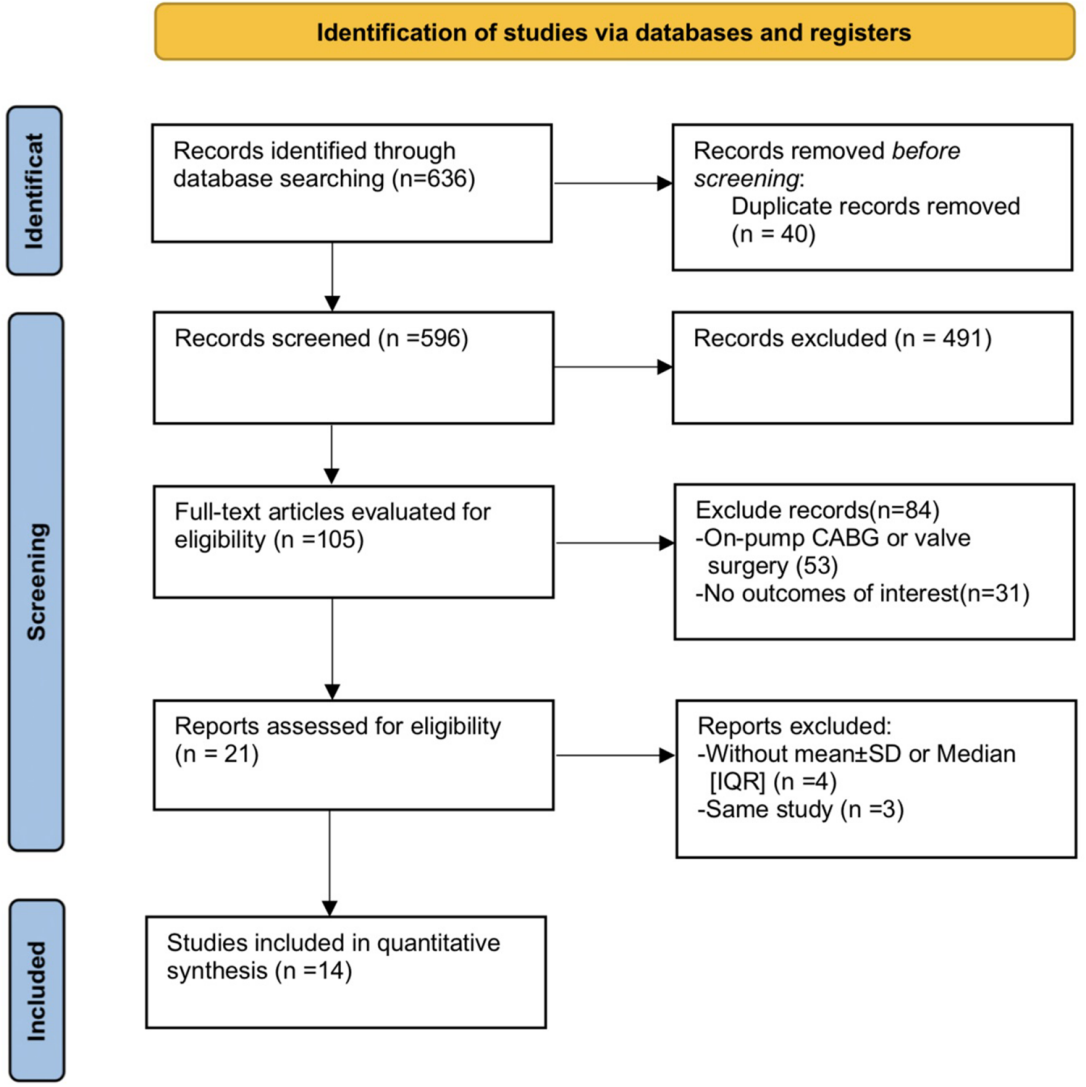
Flow chart.

We first removed any duplicate articles, leaving us with 596 qualified articles which were then further reduced to 105 for further evaluation following detailed examination of their titles and abstracts. These 105 were then further reduced to only 14 articles fulfilling all our eligibility criteria for inclusion. A flowchart describing these selections and our exclusion criteria is shown in Figure 1.

### 3.2. Included trails characteristics

A total of 14 RCTs were incorporated in the final meta-analysis, as depicted in Table 1. This study included a total of 703 samples, of which 355 were assigned to the volatile anesthetics group. Two trials documented the postoperative levels of cTnT in patients, while two articles reported the median and interquartile range (IQR) of the postoperative levels of cTnI. The cardiac troponins data collected from various trials ranged from 0 to 72 h after surgery, and the time points of peak levels in each group were recorded in Table 1. We presented the primary drugs utilized for anesthesia induction and maintenance in each group, as demonstrated in Table 1, excluding preoperative medications and muscle relaxant.

**Table 1:** Characteristics of included studies.

References	Design	*n*	Age (V/P)	Number of grafts (V/P)	Volatile group		Propofol group		cTn recorded time points(h)	cTnI/cTnT
					Induction	Maintenance	Induction	Maintenance		
Conzen 2003 (7)	RCT(SB)	20	62 (9)/65 (8)	1.3 (0.5)/1.5 (0.5)	Etomidate/sufentanil	Sevoflurane/sufentanil	Propofol/sufentanill	Propofol/sufentanil	0/3/6/12/18/24	cTnI
Mi 2003 (8)	-	26	[39–70]	2.4(-)/2.5(-)	Ketamine/midazolam/fentanyl	Isoflurane/fentanyl	Ketamine/midazolam/fentanyl	Propofol/fentanyl	4	cTnI
Kendall 2004 (9)	RCT(SB)	19	58 (7)/68 (11)	3.4 (2.5)/3 (1.7)	Etomidate/fentanyl	Isoflurane	Propofol/fentanyl	Propofol	0/3/6/12/24/48	cTnT
Guarracino 2006 (10)	RCT(TB)	112	69 (9)/69 (8)	2.5 (0.9)/2.6 (0.9)	Midazolam/fentanyl	Desflurane/fentanyl	Midazolam/fentanyl	Propofol/fentanyl	0/4/8/12	cTnI
Shan 2009 (11)	RCT(SB)	42	[43–75]	NA	Etomidate/midazolam/fentanyl	Isoflurane/fentanyl	Etomidate/midazolam/fentanyl	Propofol/fentanyl	6/12/24	cTnI
Wang 2009 (12)	RCT(SB)	60	< 75	[2–4]/[2–4]	Sevoflurane/sufentanil	Sevoflurane/sufentanil	Propofol/sufentanil	Propofol/sufentanil	6/20/28/38	cTnI
Kim 2011 (13)	RCT(SB)	94	64 (11)/65 (9)	2.7 (0.7)/2.7 (0.9)	Etomidate/remifentanil	Sevoflurane/remifentanil	Etomidate/remifentanil	Propofol/remifentanil	0/12/24	cTnI
Qi2011 (14)	RCT(SB)	60	[40–75]	NA	Midazolam/sufentanil	Sevoflurane/sufentanil	Midazolam/sufentanil	Propofol/sufentanil	0/2/4	cTnI
Tempe 2011 (15)	RCT(DB)	40	53 (8)/54 (9)	3 (1.6)/3 (1.6)	Thiopental/fentanyl	Isoflurane/fentanyl	Thiopental/fentanyl	Propofol/fentanyl	6/24	cTnT
Guerrero 2013 (16)	RCT(SB)	40	[61–73]/[62–74]	2 (1.6)/2 (1.6)	Etomidate/fentanyl	Sevoflurane/fentanyl/remi-fentanil	Etomidate/fentanyl	Propofol/fentanyl/remi-fentanil	12/24	cTnI
Ji 2013 (17)	RCT(SB)	56	62 (9)/62 (10)	2.8 (0.5)/2.8 (0.6)	Etomidate/midazolam/fentanyl	Sevoflurane/fentanyl	Etomidate/midazolam/fentanyl	Propofol/fentanyl	2/8/24	cTnI
Jiang 2013 (18)	RCT(TB)	24	57 (9)/60 (7)	2.3 (0.7)/2.1 (0.6)	Midazolam/fentanyl	Sevoflurane/midazolam/fentanyl	Midazolam/fentanyl	Propofol/midazolam/fen-tanyl	0/24/48/72	cTnI
Mroziński 2014 (19)	RCT(SB)	60	65 (9)/60 (11)	[2–4]/[1–6]	Etomidate/fentanyl	Desflurane/fentanyl	Etomidate/fentanyl	Propofol/fentanyl	18	cTnI
Guo 2017 (20)	RCT(SB)	50	< 75	[2–4]/[2–4]	Etomidate/sufentanil	Desflurane/sufentanil	Etomidate/sufentanil	Propofol/Sufentanil	6/12/24/36	cTnI

V/P, values are presented as Volatile group/Propofol group. Data are presented as mean (SD) or range: [minimum-maximum].

### 3.3. Quality assessment

**Figure 2:**
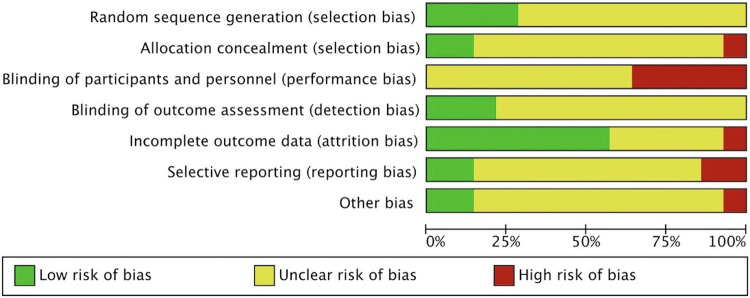
Risk of bias graph.

**Figure 3:**
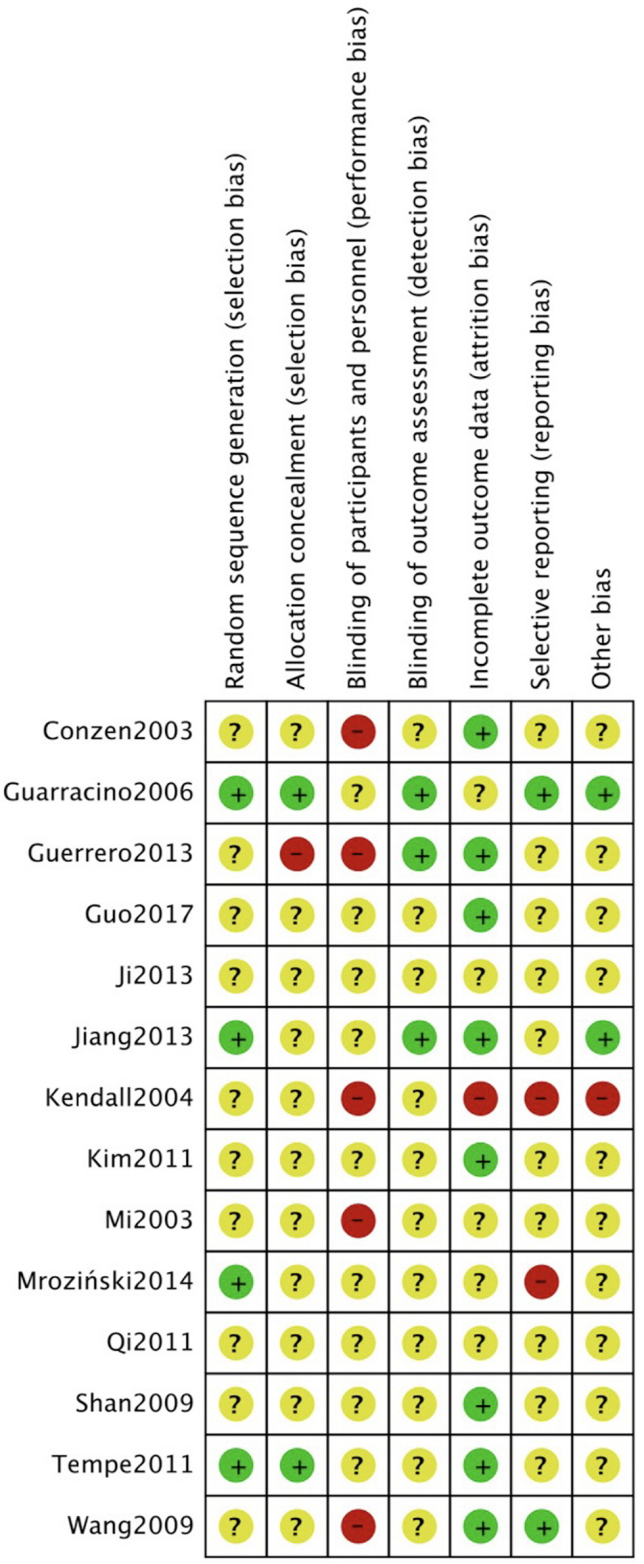
Risk of bias summary.

The risk of bias assessment for the 14 randomized controlled trials (RCTs) was conducted using the Cochrane Collaboration tool, as depicted in Figures 2, 3. (Figures 2, 3) Although most trials reported randomly assigning patients, we cautiously label those that do not specify the method of generating random sequences as “uncleared risk”. Due to the different methods of administration of volatile anesthetics and propofol, concealment, and binding allocation are relatively difficult, which also poses the main risk of bias.

### 3.4. Effects on cardioprotection

**Figure 4:**
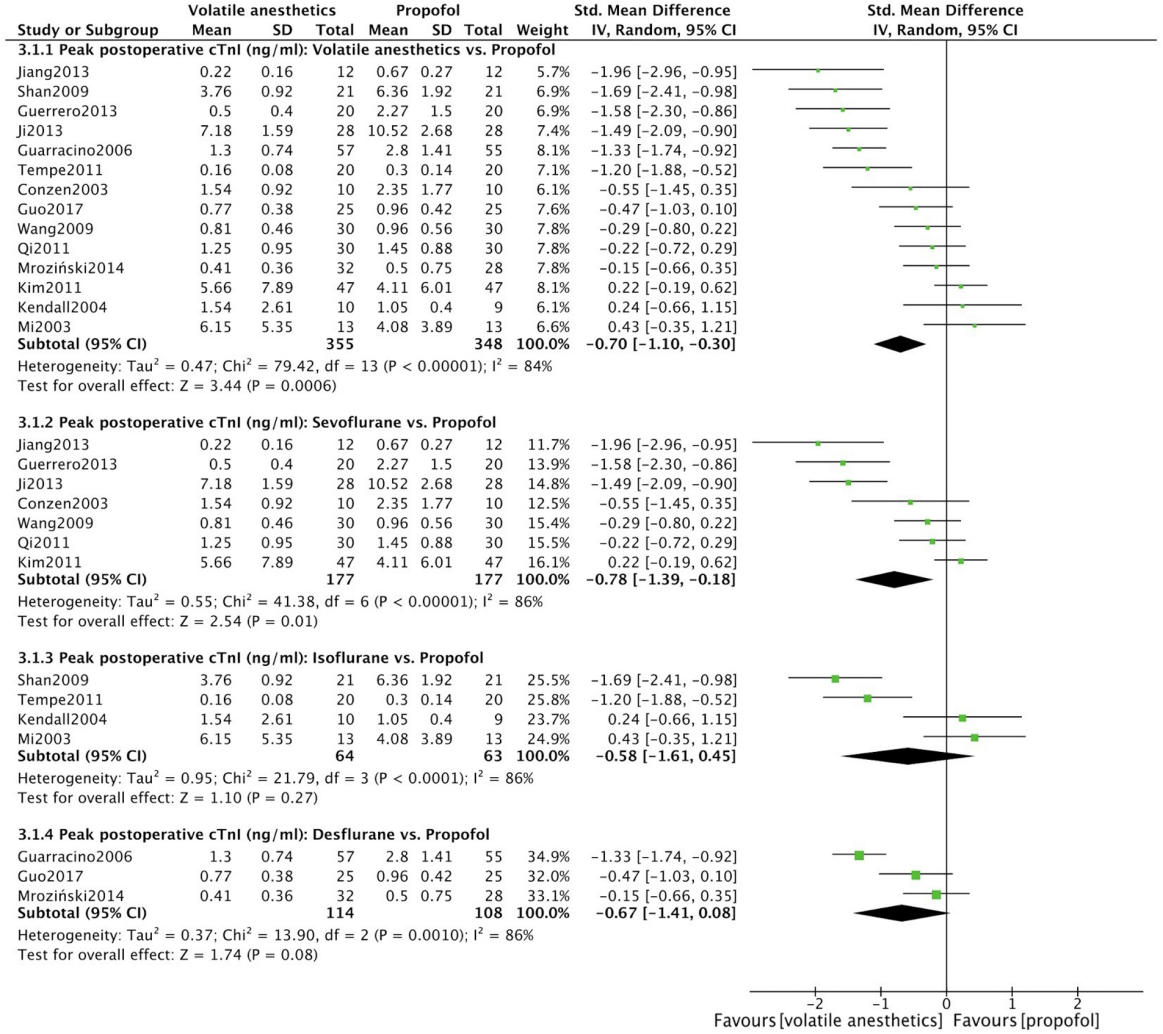
Meta analysis of postoperative peak cTnI levels in OPCAB patients between volatile anesthetic(s) group and propofol group.

As the heterogeneity test (*I*^2^ = 84%) significantly revealed inconsistency within the 14 assessed RCTs, it is recommended to interpret the results based on the random effects model rather than the fixed effects model. The meta-analysis (Figure 4) resulted in a significant outcome favoring volatile anesthetics use over propofol during OPCAB surgery concerning peak postoperative cTnI serum levels (SMD = −0.70, 95% CI: −1.10 to −0.30, *P* < .001; *I*^2^ = 84%).

The three subgroups categorized based on the different volatile anesthetics exhibit significant statistical heterogeneity (*I*^2^ > 50%), which suggests that the random effects result above should be chosen over the fixed effects model when interpreting the results. Compared to propofol, patients administered sevoflurane displayed a statistically significant reduction in postoperative cTnI serum levels (SMD = −0.78, 95% CI: −1.39 to −0.18, *P* = .01; *I*^2^ = 86%). However, neither desflurane nor isoflurane showed a similarly significant difference (Figure 4).

### 3.5. Effects on postoperative recovery

**Figure 5:**
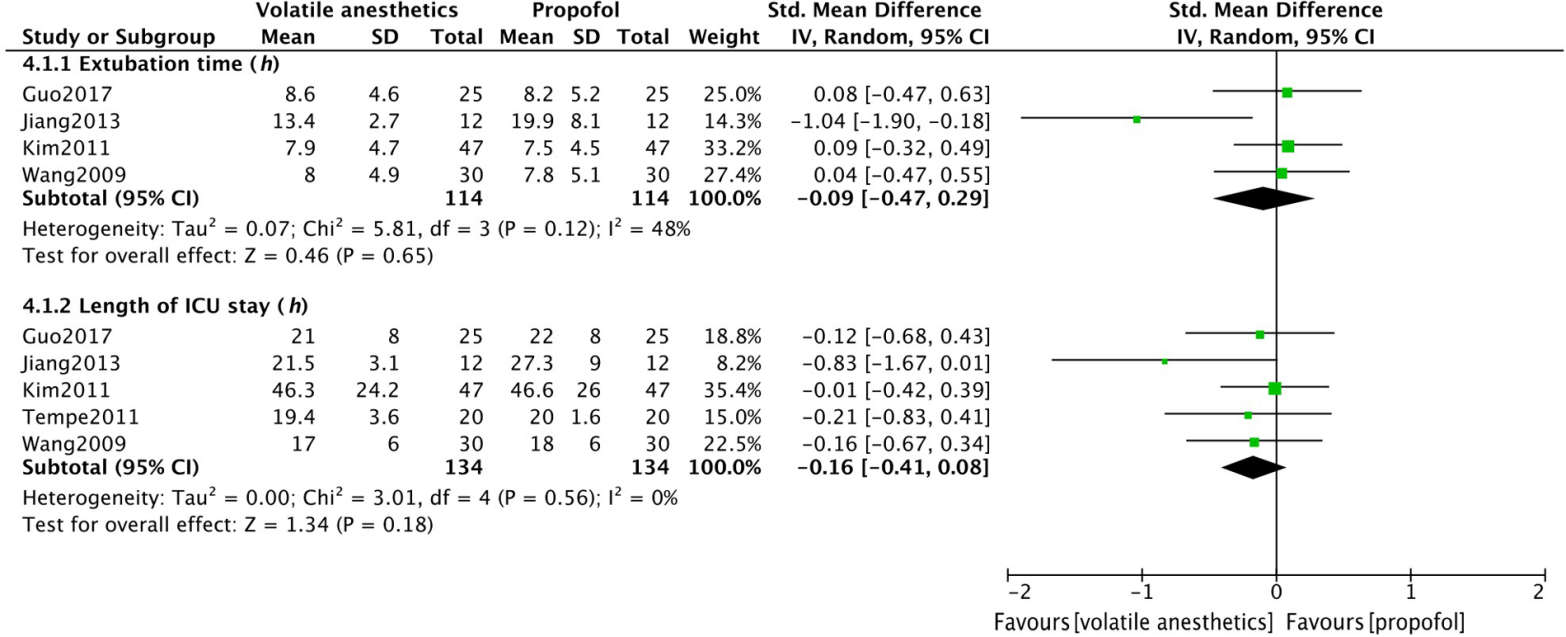
Meta analysis of postoperative extubation time and the length of ICU stay in OPCAB patients between volatile anesthetic(s) group and propofol group.

Four trials focused on postoperative extubation time, and five recorded the length of ICU stay for patients after surgery. A meta-analysis of the above results showed that volatile anesthetics could not accelerate the postoperative recovery of OPCAB patients (Figure 5). However, due to limited follow-up time after surgery, there is insufficient data for further analysis of short-term patient mortality rates.

## 4. Discussion

In 2016, Straarup and colleagues conducted a meta-analysis on the cardioprotective effects of volatile anesthetics vs. propofol in patients undergoing OPCAB (3). They posited that there wasn't sufficient evidence to affirm a significant advantage of volatile anesthetics in reducing postoperative cardiac troponin levels in OPCAB patients. Given that three of the studies included in Straarup et al.'s research presented serious issues (two of them being preconditioning trials without a direct comparison between volatile anesthetics and propofol, and one merely reporting the mean postoperative troponin levels), their study conclusions are inevitably questionable. After excluding flawed articles and incorporating recent research, including several papers from Chinese sources, our sample size increased to 703 patients. Our meta-analysis suggests that, compared to propofol, the use of volatile anesthetics during OPCAB surgery might also decrease postoperative cardiac troponin levels.

Animal studies have found that sevoflurane, desflurane, and isoflurane all protect mitochondria by modulating cyclophilin D (CypD), thereby helping to reduce myocardial ischemia-reperfusion injury (21, 22). This can help maintain cellular energy levels and prevent cell death. Many studies suggest that sevoflurane and desflurane have cardioprotective effects and reduce postoperative cardiac troponins levels for cardiac surgery (23, 24). Our subgroup analysis based on different volatile anesthetics administered also supports the hypothesis that sevoflurane may reduce postoperative cardiac troponins release. We observed that postoperative troponin levels in the desflurane group were lower than those in the propofol group, although the difference was not significant due to the limited sample size. The cardioprotective mechanisms of volatiel anesthetics are complex, and different drugs may have varying power in terms of myocardial protection (25). Contrary to conventional belief (26), the evidence included in our study could not confirm that the use of isoflurane in OPCAB surgery could considerably decrease postoperative cardiac troponins levels. Even Jeong et al. (27) found in a retrospective study of 712 patients that the mean peak postoperative cardiac troponins level was higher in the isoflurane group than in the propofol group. Therefore, to investigate the myocardial protective effects of volatile anesthetics in patients undergoing OPCAB surgery, large-scale prospective randomized controlled trials are still required, particularly for head-to-head comparisons among various volatile anesthetics.

Both sevoflurane and propofol exert cardiac protection through distinct mechanisms and their effects are dose-dependent (28–30). Research has demonstrated that high doses of propofol may provide superior cardiac protection compared to sevoflurane in patients with high-risk factors, such as severe ischemia, cardiovascular instability, and emergency or urgent surgeries (31). The studies we examined lacked consistent target levels for each drug, which may also be a reason for the different outcomes of each trial. Therefore, the optimal dosage of each drug in terms of myocardial protection is worth further exploration.

Postoperative peak cTnI level >13 ng/ml was an independent predictor of 30-day major adverse cardiovascular events and all-cause mortality after adult cardiac surgery (32, 33). In two studies included (13, 17), some patients demonstrated postoperative peak cTnI levels approaching the aforementioned cut-off value. However, both articles did not report the occurrence of serious cardiovascular events and death after surgery. Some researchers contend that the true cardioprotective effects of volatile anesthetics produce reductions of 30 to 40% if enzyme release is graphed over time (area-under-the-curve), thereby providing a more accurate estimate of perioperative myocardial injury as it precisely quantifies the extent during that specific period (34). Omran et al. believed high-sensitivity cTnI level determined 12–16 h after cardiac surgery correlated best with myocardial ischemia and a decision to repeat revascularization, while at earlier time points, the clinical decision should rather be based on electrocardiogram, echocardiographic, and hemodynamic criteria (32). Among the 14 articles included in this study, there were significant differences in the selection of time points for postoperative cTn level measurement in patients, and some even collected cTn levels at only one-time point (8, 19). One study by Wang et al. showed that postoperative peak cTnI levels occurred immediately and 72 h after surgery in two groups (18). In other articles, postoperative peak cTnI levels were mostly observed within 24 h after surgery. The results of this study show that volatile anesthetics can help reduce cardiac troponin levels after surgery, but there is no difference between the two groups in serious cardiovascular events, mortality, and postoperative recovery. In summary, for patients undergoing OPCAB surgery, postoperative peak cTnI level may not truly reflect the degree of myocardial injury, and AUC cTnI may better reflect the degree of myocardial injury (35).

The impact of volatile anesthetics and propofol on postoperative mortality rates in cardiac surgery patients remains controversial. In a multicenter randomized controlled trial conducted by Landoni et al. in 2019, which included 5,400 patients, they did not find that volatile anesthetics could reduce postoperative mortality in cardiac surgery patients (36). Similarly, in a meta-analysis published by Jiao et al. in 2019, there was no evidence to suggest that volatile anesthetics could help reduce postoperative mortality rates in cardiac surgeries, encompassing both on-pump and off-pump CABG procedures (37). Conversely, a meta-analysis published by Alice Bonanni in 2020, which exclusively included on-pump CABG surgery patients, posited that volatile anesthetics could potentially reduce the 1-year postoperative mortality rate (38). Additionally, patients in the volatile group exhibited lower postoperative cardiac troponin levels and a reduced likelihood of perioperative myocardial infarction (38). Among the 14 studies included in our analysis, only one reported a patient death within 30 days after OPCAB surgery, which occurred in the propofol group (10). In the study by Mroziński et al., no deaths were reported within one year after surgery, however, during their follow-up of patients for over 5 years, a total of 5 deaths were recorded, with 4 of them belonging to the propofol group (19). Although the use of volatile anesthetics in cardiac surgery may confer benefits in reducing postoperative cardiac troponin levels, it does not necessarily imply that using volatile anesthetics can help decrease mortality in patients undergoing cardiac surgery. The impact of the two types of drugs on postoperative mortality rates in OPCAB surgery patients remains to be further elucidated.

Due to the limited sample size, there was no statistically significant difference in extubation time and length of ICU stay between the two groups. Moreover, it should be noted that there are variations in medical standards and postoperative management strategies across different regions. Consequently, the time to extubation and the length of ICU stay for patients will inevitably differ. Results comparing postoperative recovery should be approached with caution. Further large-scale studies are needed to examine the impact of postoperative recovery and long-term survival using consistent postoperative management strategies and standardized endpoints.

Several relevant limitations of this study are worth mentioning. This study has the inherent weakness of meta-analysis. Meta-analysis can increase the power of analysis by pooling many small studies, but different clinical practices and a lack of uniform definition of some endpoints may limit the certainty of meta-analysis findings. Another problem was that heterogeneity was identified in this meta-analysis. Heterogeneity could be multifactorial, such as variance in cTnI measurement time, intraoperative anesthesia management, and different postoperative treatment strategies among different centers.

## 5. Conclusion

In conclusion, we believe that for patients undergoing OPCAB surgery, compared to propofol, volatile anesthetics, particularly sevoflurane, can help reduce cardiac troponin levels after surgery. Existing evidence is not sufficient to support the superiority of isoflurane and desflurane over propofol in myocardial protection. Whether inhalation of anesthetics can accelerate postoperative recovery in OPCAB patients and reduce mortality remains to be further studied.

## Data Availability

The original contributions presented in the study are included in the article/Supplementary Material, further inquiries can be directed to the corresponding author.
